# Changes in health-related quality of life following imprisonment in 92 women in England: a three month follow-up study

**DOI:** 10.1186/1475-9276-10-21

**Published:** 2011-05-25

**Authors:** Emma Plugge, Nick Douglas, Ray Fitzpatrick

**Affiliations:** 1 Department of Public Health, University of Oxford

## Abstract

**Background:**

Despite the considerable changes in the provision of health care to prisoners in the UK there is little published literature that attempts to examine broader aspects of health and the impact of imprisonment on these, focusing instead on disease specific areas. This is surprising given that one of the main drivers behind the changes was the need for improvements in the quality of care; examining changes in health outcomes should be an important part of monitoring service developments. This study assessed the health-related quality of life of women on entry into prison and examined changes during a period of three months imprisonment.

**Methods:**

This was a prospective longitudinal study involving 505 women prisoners in England. The SF-36 was contained within a questionnaire designed to examine many aspects of imprisoned women's health. Participants completed this questionnaire within 72 hours of entering prison. The researchers followed up all participants who were still imprisoned three months later.

**Results:**

The study achieved good response rates: 82% of women agreed to participate initially (n = 505), and 93% of those still imprisoned participating three months later (n = 112). At prison entry, women prisoners have lower mental component summary score (MCS) and physical component summary score (PCS) compared to women within the general population. The mental well-being of those 112 women still imprisoned after three months improved over this period of imprisonment, although remained poorer than that of the general population. The PCS did not improve significantly and remained significantly lower than that of the general population. Multivariate analyses showed that the only independent predictor of change in component score was the score at baseline.

**Conclusions:**

The results highlight the poor health-related quality of life of women prisoners and highlight the scale of the challenge faced by those providing health care to prisoners. They also draw attention to the major health disadvantages of women offenders compared to women in general. While recent reforms may improve health services for prisoners, broader inequalities in the health of women are a more complex challenge.

## Background

There are approximately 85,000 prisoners in England and Wales and a small but increasing proportion of them are women; women prisoners number about 4,300 [1]. Prisoners in the United Kingdom (UK) are a socially excluded group. Compared with the general population, prisoners are 13 times as likely to have been in care as a child, 13 times as likely to be unemployed and 10 times as likely to have been a regular truant. Their basic skills are very likely to be poor; 80 per cent have the writing skills, 65 per cent the numeracy skills and 50 per cent the reading skills at or below the level of an 11-year-old child [2]. Women prisoners are particularly disadvantaged in a system designed for men by men [3]. They are not imprisoned for violent crime but for acquisitive crime; much of this is drug related, committed either by drug users to fund their habit (theft and handling, burglary or robbery) or by women involved in the trafficking and sale of drugs (drug offences) [4]. It is therefore not surprising that substance use is a considerable health problem for imprisoned women [5].

There is also evidence indicating a number of other important health issues for women prisoners: mental health problems [6], blood borne virus infections [7,8], sexually transmitted infections [9], cervical dysplasia [10,11] and pregnancy complications [12,13]. Even though there is this apparent high level of need, women prisoners are more likely to experience reduced access to health services in the community [2].

Despite the increasing amount of evidence on imprisoned women's health and the considerable changes in the provision of health care to prisoners in the UK which have resulted in the transfer of health services from the Prison Service to the National Health Service [14], there is little published literature that attempts to examine broader aspects of health and the impact of imprisonment on this. This is surprising given that one of the main drivers behind the changes was the recognised need for improvements in the quality of care [14]. Examining changes in health outcomes should be an important part of monitoring the progress of these service changes. However, the current research tends to focus on disease specific areas rather than using quality of life measures to gain a fuller picture of these women's health. Furthermore, most of the studies are cross sectional or case-control in design and thus take only a snapshot of women's health at one point during imprisonment. There are however instruments that are able not only capture broader aspects of well-being but are also sensitive to change over time. The short form 36 (SF-36) is such an instrument which not only is well validated [15] but has demonstrated its usefulness in a female prison population in England [16]. The SF-36 therefore, as a health outcome measure, has the potential to contribute to the monitoring of health service changes with prisons.

The overall aim of this study was to examine changes in the health related quality of life of women prisoners during a period of three months imprisonment.

## Methods

### Design and Participants

We conducted a secondary analysis of the study data from one of the largest studies examining the health of women prisoners in England and Wales [17]. This study took place in women's prisons in England and was completed in 2006. The two researchers (ND and EP) were based in one of the two prisons in which recruitment was taking place. All women received into these two prisons on a pre-specified study day during the recruitment period were eligible to participate; the recruitment took six months. Those posing a security threat or who were severely mentally ill were excluded; a total of 11 women were excluded. Women were given written information and a verbal explanation about the study. The researchers obtained written consent from participants before giving them the questionnaire to complete in private. All women were recruited within 72 hours of coming into prison. The sample size was calculated using the computer package 'Power and Precision' [18] and used data from the pilot study. A final sample size at three months of 110 women was necessary but statistics from the Home Office [4] indicated that only one third of female prisoners would still be imprisoned then. Assuming that at least 30% of the original sample would not agree or be unable to participate at three months we aimed to recruit 500 women.

The researchers were available to administer the questionnaire if the participant requested. The researchers then followed up all participants who were still imprisoned three months later and invited them to participate again and complete a further questionnaire. This study examines data only on those women who were still imprisoned three months later.

### Measures

The SF-36 (version 2) was contained within a questionnaire which also asked about health related behaviours such as alcohol consumption, and about personal characteristics such as age. The questions about health related behaviours were from the Oxford Healthy Lifestyle Survey. This postal survey consistently achieved high response and completion rates across all sectors of the population, regardless of socio-economic status or ethnicity [19].

### Analysis

We analysed the information from completed questionnaires of women who remained in prison at three months using SPSSv13. We compared data on demographic information for the study sample with the published data for all women prisoners which were obtained from the Home Office published statistics on Women and the Criminal Justice System [4]. We examined differences between those women prisoners for whom we had data at three months and those women prisoners who were still imprisoned at three months but did not provide data either because they did not participate again or because their data was incomplete.

We calculated the scores for each of the eight dimensions with 95% confidence intervals, and the physical and mental component summary scores (PCS and MCS) for the SF-36 according to the developers manual [15,20]. Changes in dimension and summary scores during imprisonment were also investigated. Changes in summary scores for different groups of prisoners were assessed using the paired t-test for means. For example, we assessed changes in age by comparing those aged 30 years or more to those aged under 30; 30 was used as this was the modal age. Significance was assumed at p < 0.05. Multiple linear regression was used to explore these relationships further; all variables with p < 0.20 in the univariate analysis were entered into the regression model.

## Results

### The sample

Five hundred and five out of 613 women approached completed the questionnaire giving a response rate of 82.4%. Of the original 505 participants, 120 were still in prison three months later and were therefore the focus of this study. Of these, 112 (93%) participated again; complete data on SF-36 scores on entry into prison and three months later was available for 92 (82%) participants. When compared to all women prisoners in England, the study sample of 112 women were less likely to be white (58% v 71%) and more likely to have stayed in school after the age of 16 years (39% v 26%). There were no significant differences in terms of age; the proportion of women in this sample aged 21 to 39 years was 64% compared to the 69% of all women prisoners in England. The mean (SD) age of these women was 33.5 (10.4) years, range 19 to 72 years.

There were no significant differences between the 92 women who contributed data at three months and the 28 women who did not; these findings are reported in Table 1. There was no significant difference in age; the mean (SD) age of those with data was 33.0 (10.2) years compared to 35.8 (11.7) years for those without (p = 0.29).

**Table 1:** Comparison of women who were still imprisoned at three months: those who complete data & those with missing data.

	Women with complete data at 3 months %	Women with missing data at 3 months %	Difference in % (95% confidence interval)	P value
Self reported ethnicity: white	60.7	57.9	2.8 (-18.8 to 26.4)	0.82

Has children aged under 16 years	42.5	50.0	7.5 (-15.9 to 30.6)	0.75

Left school aged 16 years or less	61.8	58.8	3.0 (-19.2 to 27.7)	0.82

Was unemployed before coming into prison	68.9	73.7	4.8 (-19.3 to 22.5)	0.89

Has been in prison before	47.6	47.1	0.6 (-23.7 to 23.9)	0.97

Drinks more than 3 units each day	38.0	35.0	3.0 (-20.6 to 22.8)	0.79

Injected drugs in week prior to imprisonment	9.9	10.0	0.1 (-10.6 to 20.7)	0.99

Had a longstanding illness before prison	84.9	78.9	5.9 (-9.5 to 29.0)	0.77

### Health-related quality of life on entry to prison and changes during imprisonment

The results in Table 2 show the eight dimension scores and the two summary scores of the sample of women prisoners in this study on entry into prison and again three months later. Of note, both the MCS of 35.5 and PCS of 44.3 on entry to prison compared unfavourably with a MCS of 48.9 and PCS of 49.1 for women in the general population in England [20].

**Table 2:** Comparison of SF-36 dimension and summary scores on entry into prison and three months later.

	Women still in prison at three months, n = 92		Difference [95% confidence interval]	Significance
			
	On entry to prison Mean (sd)	Three months after imprisonment Mean (sd)		
physical functioning	72.6 (29.1)	80.6 (25.5)	8.0 [2.0 to 13.0]	0.002

role physical	69.9 (31.1)	74.9 (28.6)	5.0 [-1.5 to 11.5]	0.127

role emotional	56.1 (35.8)	64.6 (32.3)	8.6 [1.8 to 15.4]	0.014

social functioning	49.1 (32.2)	58.7 (28.1)	9.6 [2.8 to 16.4]	0.006

mental health	45.6 (25.1)	51.6 (23.3)	6.0 [1.6 to 10.4]	0.008

energy/vitality	42.0 (25.1)	48.1 (23.6)	6.1 [0.7 to 11.4]	0.027

pain	58.0 (29.9)	63.4 (27.4)	5.5 [-1.0 to 11.9]	0.098

general health perception	49.8 (25.4)	58.2 (23.0)	8.4 [3.5 to 13.2]	0.001

Mental Component Summary score	35.5 (14.7)	40.1 (14.0)	4.6 [1.7 to 7.5]	0.002

Physical Component Summary score	44.3 (12.0)	46.3 (12.2)	1.9 [-0.6 to 4.4]	0.131

Changes in health-related quality of life showed that there was significant improvement following imprisonment in several domains. In those women still in at three months with complete data, there was a statistically significant improvement in physical functioning, role emotional, social functioning, mental health, energy, general health perception and the mental component summary score.

Univariate analysis of changes in MCS and PCS scores within particular groups suggested that greater change occurred in some groups than others. The MCS improved in those who had been in prison before and those who had injected drugs in the week prior to imprisonment. The PCS improved in women who were white or who left school at 16 years or less, or were unemployed before imprisonment or who had been in prison before or who had injected drugs in the week prior to imprisonment. However, the results of the subsequent multivariate analyses showed that the only independent predictor of change in component score was the score at baseline. These findings are detailed in Tables 3, 4, 5 and 6.

**Table 3:** Change in mental component summary score of SF36 (MCS) over three months' imprisonment: results of univariate analysis.

		N	MCS on entry to prison Mean (sd)	MCS three months after imprisonment Mean (sd)	Mean change in MCS	p value
Aged 30 years or more	Yes	53	38.1 (15.7)	41.5 (14.1)	3.4	0.34

	No	39	31.9 (12.5)	38.1 (13.9)	6.2	

Self reported ethnicity: white	Yes	54	34.8 (14.5)	40.7 (14.3)	5.9	0.40

	No	35	35.5 (15.2)	38.8 (13.9)	3.3	

Has children aged under 16 years	Yes	37	36.7 (16.2)	40.5 (15.6)	3.8	0.55

	No	50	38.0 (15.0)	42.6 (13.7)	4.6	

Left school aged 16 years or less	Yes	55	32.9 (14.7)	39.6 (14.1)	6.6	0.11

	No	34	39.2 (14.1)	40.9 (14.1)	1.6	

Unemployed before coming into prison	Yes	62	33.4 (15.1)	39.9 (14.3)	6.5	0.052

	No	28	39.6 (13.2)	39.9 (14.1)	0.3	

Has been in prison before	Yes	40	33.8 (15.5)	41.2 (13.5)	7.4	0.047

	No	44	37.5 (13.6)	38.9 (14.7)	1.4	

Drinks more than 3 units each day	Yes	35	31.9 (15.0)	39.3 (11.3)	7.3	0.23

	No	57	37.5 (14.4)	40.7 (15.7)	3.2	

Injecting drug use in week prior to imprisonment	Yes	9	23.7 (11.5)	40.1 (16.9)	16.5	0.007

	No	82	36.6 (14.5)	39.9 (13.9)	3.4	

Longstanding illness before prison	Yes	73	32.7 (13.5)	38.0 (14.0)	5.4	0.062

	No	13	51.9 (9.5)	49.5 (11.0)	-2.4	

**Table 4:** Factors examined for association with a change in mental component summary score of SF36 (MCS): results of multivariate analysis, n = 78

Predictor	Coefficient B	95% confidence interval for B	P value
MCS on entry into prison	-0.44	-0.65 to -0.22	<0.001

Left school aged 16 years or less	0.12	-6.63 to 6.87	0.92

Unemployed before coming into prison	3.52	-3.04 to 10.07	0.29

Has been in prison before	3.11	-3.26 to 9.49	0.33

Intravenous drug use in week prior to imprisonment	5.28	-4.50 to 15.06	0.29

Longstanding illness before prison	-3.93	-12.72 to 4.86	0.38

**Table 5:** Change in physical component summary score of SF36 (PCS) over three months' imprisonment: results of univariate analysis

		N	PCS on entry to prison Mean (sd)	PCS three months after imprisonment Mean (sd)	Mean change in PCS	p value
Aged 30 years or more	Yes	53	44.5 (12.5)	44.8 (12.5)	0.4	0.15
	No	39	44.1 (11.5)	48.1 (11.7)	4.0	

Self reported ethnicity: white	Yes	54	44.4 (11.3)	48.4 (10.2)	3.9	0.044
	No	35	44.4 (13.4)	43.0 (14.6)	-1.3	

Has children aged under 16	Yes	37	46.8 (10.9)	50.2 (8.9)	3.4	0.66
years	No	50	42.9 (14.2)	42.6 (13.7)	0.4	

Left school aged 16 years or	Yes	55	43.4 (12.2)	47.6 (12.8)	4.2	0.019
less	No	34	45.8 (11.7)	44.2 (11.1)	-1.5	

Unemployed before coming	Yes	62	41.9 (12.5)	45.9 (12.5)	3.9	0.019
into prison	No	28	50.4 (8.4)	47.9 (11.5)	-2.5	

Has been in prison before	Yes	40	40.5 (12.8)	46.1 (13.5)	5.6	0.004
	No	44	48.7 (9.5)	46.4 (10.7)	-2.3	

Drinks more than 3 units each	Yes	35	42.3 (12.9)	45.8 (14.9)	3.4	0.30
day	No	57	45.8 (11.4)	46.6 (10.5)	0.9	

Injecting drug use in week	Yes	9	35.9 (10.7)	47.8 (16.0)	11.9	0.008
prior to imprisonment	No	82	45.4 (11.7)	46.2 (11.8)	0.8	

Longstanding illness before	Yes	73	44.5 (11.5)	46.4 (11.8)	1.9	0.59
prison	No	13	49.8 (9.5)	50.3 (9.3)	0.5	

**Table 6:** Factors examined for association with a change in physical component summary score of SF36 (PCS): results of multivariate analysis, n = 81

Predictor	Coefficient B	95% confidence interval for B	P value
PCS on entry into prison	-0.45	-0.67 to -0.24	<0.001

Age	-0.17	-0.39 to 0.07	0.16

Self reported ethnicity: white	4.56	-0.43 to 9.55	0.073

Left school aged 16 years or less	3.20	-2.58 to 8.98	0.27

Unemployed before coming into prison	-0.27	-5.98 to 5.44	0.93

Has been in prison before	0.52	-5.15 to 6.20	0.85

Intravenous drug use in week prior to imprisonment	5.69	-2.65 to 14.04	0.18

## Discussion

This study presents unique longitudinal data on the health-related quality of life of women prisoners in England. The results highlight the poor physical, psychological and social health of women prisoners but also show that their mental well-being improved over the three months of imprisonment. Physical health as measured by the PCS did not improve significantly and remained lower than that of the general population.

The design and conduct of the study had several advantages. The researchers met with the women on more than one occasion and this is likely to have contributed to good data quality by helping them gain the confidence and trust of the participants. The study achieved good response rates with 82% of women agreeing to participate initially, and 93% of those still imprisoned participating three months later. However, we acknowledge that 92 women comprise a small sample of the total female prison population. Analysis of specific demographic information on participants suggested that this sample did differ from the general population of women prisoners in England. Although similar in age, this sample was less likely to be white and more likely to have stayed in education beyond the age of 16 years. However, it may be that this sample was similar to women who stay in prison for at least three months but no data was available from HM Prison Service to explore this. A further issue regarding the generalizability of the findings is that these women were only followed up for three months and it is therefore not possible to know how they differed from women who were imprisoned for longer or whether the changes would be sustained. It would be important for future research studies to follow up women for a longer period.

The SF-36 was an appropriate instrument to measure health-related quality of life in this population. It provides information on a number of aspects of well-being: physical, mental, psychological and social. It is sensitive to change over the short period of three months and suitable in a general, not solely a patient, population [21]. As noted, previous research has also demonstrated its usefulness in a female prison population in England [16]. However, it has not been widely used with prison populations and this is the first study to have used the SF-36 to monitor women prisoners' health status over time.

On entry into prison, women prisoners had lower summary scores compared to than those in the general population. The mean MCS was 35.5; only 14.3% of the general female population have a score of 36 or less [22]. The mean PCS was 44.3; only 22.8% of females have a score of 45 or less [22]. A small number of published studies have reported the SF-36 dimension and summary scores for prisoners. These studies report that scores for several dimensions were significantly lower than for the general population [16,23-25]. The UK study found that the dimensions of social functioning, mental health, energy/vitality, pain and general health perception were all significantly lower than scores for the general population [16]. An Australian study looking at prisoners infected with hepatitis C found that they had lower scores in four of the eight domains: social functioning, role emotional, mental health and general health [23].

The mental well-being of these women, as measured by the SF-36, improved over the three months of imprisonment. The reasons for the improvement in mental well-being are unclear. For those who have visited prison and experienced the unique environment - restrictive, lacking comfort or the presence of loved ones- it is difficult to see why there should be any improvement in health. For many people, this could only damage their health. However, it is perhaps an indictment of the lives women prisoners lead in community that such an environment is experienced as health promoting; for women who are homeless in the community perhaps enforced containment is preferable, and for those who live in fear of violence, a locked cell door provides security. Furthermore women may benefit from the shelter, regular meals and reduction in alcohol and/or drug consumption that prison affords. Improved access to health services may be another reason for the change; women may be able to access addiction and mental health services in prison which they have been unable to do in the community. Of course the change may not be attributable to any positive effect of imprisonment, it might simply be because these women are adapting to life in prison in the way that people adapt to other stressors such as bereavement or the diagnosis of a life threatening illness [26,27]. Future qualitative research would be important in exploring this area in more depth.

These findings contrast with an Australian study which examined change in psychological health in women prisoners using the 12 item General Health Questionnaire [28]. The investigators followed women up after four months of imprisonment and found no statistically significant change in GHQ-12 score. However, this may in part be attributable to the fact that the study design used a cross section of all those in prison at a given time. It is likely that many would have been in prison some time and therefore any initial changes on prison entry would not have been captured. The difference in findings may also be related to the differences in prison regimes in the two countries which have different impacts on women's mental health.

## Conclusions

These findings highlight the scale of the challenge faced by those providing health care to prisoners and underline the need to address the health problems of women prisoners. The results also draw attention to the major health disadvantages of women offenders compared to women in general that were likely to exist prior to imprisonment. While recent reforms may improve health services for prisoners, broader inequalities in the health of women are a larger and more complex challenge.

## Competing interests

The authors declare that they have no competing interests.

## Authors' contributions

EP and RF designed the study. EP and ND collected and analysed the data. EP, ND and RF interpreted the data. EP wrote the first draft of the paper and ND and RF critically reviewed this and contributed substantially to all redrafts. All authors read and approved the final manuscript.
